# 

**Published:** 1858-06

**Authors:** 


### CONTENTS.

### ORIGINAL ESSAYS AND COMMUNICATIONS.

A Few Notes concerning the Progress of Dental Surgery, By GEO. H. PERINE, N. Y.....361
Functions of the Nervous System, By PROF. C. B. CHAPMAN,..........368
Vent Filling, By W. H. ATKINSON,..........371
"B" Noticed,..........372
Cheoplasty,..........375
Vicarious Menstruation following the Extraction of a Tooth, By PROF. J. RICHARDSON,..377
Professional Character, By DR. F. S. SLOSSON,..........378

### PROCEEDINGS OF SOCIETIES.

Proceedings of the Third Annual Meeting of the Michigan Dental Association,..........385
The New York Dental Association,..........394
Dental Convention of Northern Ohio,..........402

### SELECTIONS.

Lectures by Dr. Gladstone, F. R. S.,..........425
Popular Essays on Dentistry, By H. E. PEEBLES, D. D. S., St. Louis,..........341
On Clasps. By C. W. SPALDING, D. D. S.,..........433
Dr. Piggot's Valedictory,..........435
Amalgam,..........440
MISCELLANEOUS..........441
EDITORIAL,..........460

